# Protective efficacy of *Toxoplasma gondii*calcium-dependent protein kinase 1 (TgCDPK1) adjuvated with recombinant IL-15 and IL-21 against experimental toxoplasmosis in mice

**DOI:** 10.1186/1471-2334-14-487

**Published:** 2014-09-06

**Authors:** Jia Chen, Zhong-Yuan Li, Si-Yang Huang, Eskild Petersen, Hui-Qun Song, Dong-Hui Zhou, Xing-Quan Zhu

**Affiliations:** State Key Laboratory of Veterinary Etiological Biology, Key Laboratory of Veterinary Parasitology of Gansu Province, Lanzhou Veterinary Research Institute, Chinese Academy of Agricultural Sciences, Lanzhou, Gansu Province 730046 P.R. China; College of Animal Science and Veterinary Medicine, Heilongjiang Bayi Agricultural University, Daqing, Heilongjiang Province 163319 P.R. China; Department of Infectious Diseases, Clinical Institute, and Institute of Medical Microbiology and Immunology, Faculty of Health Sciences, Aarhus University, Aarhus, Denmark

**Keywords:** *Toxoplasma gondii*, Toxoplasmosis, TgCDPK1, pVAX-IL-15-IL-21, DNA vaccine, Protective immunity

## Abstract

**Background:**

*Toxoplasma gondii* can infect all warm-blooded animals including humans. Infection with *T. gondii* is probably the leading cause of posterior uveitis in humans and the most comment route of transmission is raw and undercooked meat from infected animals. *T. gondii* calcium-dependent protein kinase 1 (TgCDPK1) plays a critical role in direct parasite motility, host-cell invasion, and egress.

**Methods:**

We constructed a DNA vaccine expressing TgCDPK1 inserted into eukaryotic expression vector pVAX I and evaluated the immune protection induced by pVAX-CDPK1 in Kunming mice. Mice immunized with pVAX-CDPK1 intramuscularly and/or with a plasmid encoding IL-15 and IL-21 (pVAX-IL-21-IL-15). The immune responses were analyzed including lymphoproliferative assay, cytokine, antibody measurements, lymphocyte surface markers by flow cytometry and protective efficacy were measured as survival and cysts numbers after challenge 1 to 2 months post vaccination.

**Results:**

Immunization with pVAX-CDPK1 or pVAX-IL-21-IL-15 alone developed strong humoral responses and Th1 type cellular immune responses, and the significantly (*P* < 0.05) increase of both the percentages of CD4+ and CD8+ T cells compared with all the controls (blank control, PBS, and pVAX). Co-injection of pVAX-IL-21-IL-15 significantly increased humoral and cellular immune responses compared to the group of pVAX-CDPK1 or pVAX-IL-21-IL-15. Challenge experiments showed that co-administration of pVAX-IL-21-IL-15 and pVAX-CDPK1 significantly (*P* < 0.05) increased survival time (19.2 ± 5.1 days) compared with pVAX-CDPK1 (17.3 ± 4.3 days) or pVAX-IL-21-IL-15 (12.0 ± 2.0 days) alone, and pVAX-IL-21-IL-15 + pVAX-CDPK1 significantly reduced the number of brain cysts (72.7%) in contrast to pVAX-ROP13 (45.7%) or pVAX-IL-21-IL-15 alone (43.6%).

**Conclusions:**

TgCDPK1 is identified to be a promising vaccine candidate for inducing a strong humoral and cellular response against *T. gondii* infection, and thus synergistic of mIL-21 and mIL-15 can induce non-specific immune responses, but also facilitate specific humoral as well as cellular immune responses elicited by DNA vaccine against acute and chronic *T. gondii* infection in mice.

**Electronic supplementary material:**

The online version of this article (doi:10.1186/1471-2334-14-487) contains supplementary material, which is available to authorized users.

## Background

*Toxoplasma gondii* can invade a wide range of vertebrate hosts including humans, leading to a variety of clinical infections in humans [1–4]. *T. gondii* infection pose serious public safety issues [5, 6]. *T. gondii* can also cause abortion in all types of livestock, such as sheep and goats, and infected meat can serve as a source of transmission to humans [7–9].

No drugs which effectively eliminate the parasite are available, so the current development of an effective vaccine against *T. gondii* infection is a promising alternative to control toxoplasmosis in animals, and also an effective vaccine preventing infection in animals used for human consumption would block the main transmission route to humans [10, 11]. Although several types of vaccineshave been developed including genetically engineering vaccines, subunit vaccines, especially, a live and attenuated vaccine of *T. gondii* S48 strain named ToxoVax has been licensed and used in farm animals, but it has limitations of poor efficacy or biosafety concerns [10, 11]. Most efforts have been made on DNA vaccines due to their capacity to induce a Th1-type immune response including a strong CD8+ cytotoxic T-lymphocyte (CTL) response [12–14].

A group of plant-like calcium-dependent protein kinases (CDPKs), belonging to a superfamily of kinases, are in charge of the calcium signaling cascades of plants, and some ciliates [15]. In apicomplexans, CDPKs have been implicated in calcium-signal transduction involving in some events such as gliding motility or egress [16]. *T. gondii* CDPK1 protein is conserved among apicomplexans, involved in important biological function, including the regulation of the parasite’s life cycle at stages dependent on microneme secretion, and it is recognized as the key regulator of calcium dependent exocytosis and acts in calcium-dependent secretion of specialized organelles called micronemes, which play a critical role in direct parasite motility, host-cell invasion, and egress [17], but also CDPKs have been identified in plants, ciliates and apicomplexans but not expressed by mammals, which represents validated target that may be exploitable for vaccine candidate against *T. gondii*.

Improvement of the efficacy of DNA vaccines may be achieved by administration of cytokines as adjuvants. Recently, genetic adjuvants using the cytokines with the synergy of IL-21 and IL-15 have been demonstrated to induce enhanced protective immunity in animal models against infectious disease [18–20]. Also, in our previous studies, we have found that co-administration of IL-21 and IL-15 could be used as adjuvants and boost antigen-specific humoral as well as Th1 cellular immune responses induced by DNA vaccine against *T. gondii* infection [21]. In this study, we constructed a eukaryotic plasmid, pVAX-CDPK1, and examined the immunogenicity, and protective immune effect of this DNA vaccine in Kunming mice against *T. gondii* infection. Co-administration of eukaryotic plasmid encoding both IL-21 and IL-15 with pVAX-CDPK1 was used as adjuvants to enhance the Th1 immune response and increase the of protective *T. gondii* immunity.

## Methods

### Mice and parasites

Six to eight week old female Kunming mice were purchased from Lanzhou University Laboratory Animal Center (Lanzhou, China). All mice used for the experiments were raised and handled in strict accordance with the Good Animal Practice requirements of the Animal Ethics Procedures and Guidelines of the People’s Republic of China. This study was approved by the Animal Ethics Committee of Lanzhou Veterinary Research Institute, Chinese Academy of Agricultural Sciences (Approval No. LVRIAEC2011-012).

Tachyzoites of the highly virulent RH strain of *T. gondii* were preserved in our laboratory (Laboratory of Parasitology, Lanzhou Veterinary Research Institute, Chinese Academy of Agricultural Sciences), and harvested from the peritoneal fluid of mice, washed by centrifugation, and then suspended in sterile phosphate-buffered saline (PBS). The cysts of the PRU strain were obtained from the brains of orally infected Kunming mice, and maintained by monthly passage.

### Preparation of *Toxoplasma*lysate antigen (TLA)

Purified tachyzoites of *T. gondii* RH strain were disrupted by three cycles of freezing at -20°C and thawing, and then sonicated on ice at 60 W/s. The prepared cellular lysate was centrifuged for 30 min at 10, 000 × *g* at 4°C, and the supernatants were pooled, sterile filtered with 0.2 μm sterile nitrocellulose filters (Sartorius). Finally, the TLA concentration was determined via the Bradford method using bovine serum albumin (BSA) as the standard, aliquoted and stored at -70°C until use.

### Construction of DNA vaccine plasmid

Total RNA was prepared from the tachyzoites of RH strain using TRIzol reagent (Invitrogen) according to the manufacturer’s instructions, and the coding sequences of TgCDPK1 (1,749 bp, GenBank accession no. AF333958.1) were obtained by RT-PCR amplification from total RNA using designed specific primers (forward primer: 5′-CGG*GGTACC*ATGGGGCAGCAGGAAAGCAC-3′, reverse primer: 5′-GC*TCTAGA*TTAGTTTCCGCAGAGCTTCAAGA-3′), in which *Kpn* I and *Xba* I restriction sites were introduced and underlined. Followed by ligation the obtained RT-PCR product with pMD18-T vector (TaKaRa, China), the TgCDPK1 fragment cleaved from pMD-CDPK1 by *Kpn* I/*Xba* I were subcloned into the corresponding sites of pVAX I (Invitrogen) using T_4_ DNA ligase and generated plasmid pVAX-CDPK1. All recombinant plasmids were propagated in *Escherichia coli* DH5α and confirmed by specific PCR, restriction analysis and DNA sequencing.

The recombinant plasmids were purified from transformed *Escherichia coli* DH5α cells by anion exchange chromatography (EndoFree plasmid giga kit, Qiagen Sciences, Maryland, USA) following the manufacturer’s instructions, dissolved in sterile endotoxin-free TE buffer and stored at -20°C until use. Plasmid pVAX-IL-21-IL-15 was prepared and preserved previously in our laboratory. The concentration of recombinant plasmids was determined by spectrophotometer at OD_260_ and OD_280_.

### Expression of pVAX-CDPK1 plasmid in vitro

The recombinant plasmid pVAX-CDPK1 transfection into Marc-145 cells was performed using lipofectamine™ 2000 reagent (Invitrogen) as instructed by the manufacturer. In brief, forty eight hours post-transfection, cells were processed for indirect immunefluorescence assay (IFA) followed by incubation with goat anti-*T. gondii* tachyzoites polyclonal antiserum and a FITC-labeled donkey-anti-goat IgG antibody (Proteintech Group Inc., Chicago, USA). The specific fluorescence was examined through a Zeiss Axioplan fluorescence microscope (Carl Zeiss, Germany). Marc-145 cells transfected with empty pVAX I served as the negative control.

### DNA immunization and challenge infection

Six groups (35 mice in each group) were intramuscularly injected twice at 2-week intervals in three immunizations (at weeks 0, 2 and 4) with 100 μg pVAX-CDPK1 DNA in 100 μl sterile PBS, 100 μg pVAX-CDPK1 + pVAX-IL-21-IL-15 DNA in 100 μl sterile PBS, 100 μg pVAX/IL-21/IL-15 DNA in 100 μl sterile PBS, 100 μg the empty vector pVAX, PBS (100 μl/each), respectively, and one group of mice was not inoculated to constitute blank control. Blood were collected at 0, 2, 4 and 6 weeks to analyze for specific antibodies. Splenocytes were aseptically harvested for lymphocyte proliferation assay, cytokine measurements, and flow cytometric analysis. This analysis was performed in three independent experiments.

Two weeks after the last immunization, 15 mice per group were challenged intraperitoneally (IP) with 1 × 10^3^ tachyzoites of the RH strain. The survival time for each mouse and the percentages of mice survived were recorded until a fatal outcome for all animals. In addition, 10 mice per group were inoculated orally with 20 cysts of the PRU strain at day 14th after the third immunization, and observed mice daily for mortality. Four weeks after the PRU strain challenge, surviving mice were sacrificed and their brains were removed. Each brain was homogenized in 1 ml PBS. The mean number of cysts per brain was determined by counting three samples of 10 μl aliquots of each homogenized brain under an optical microscope. This analysis was performed in three independent experiments.

### Antibody analysis

Levels of IgG, IgG1 and IgG2a antibody in serum were determined by ELISA using SBA Clonotyping System-HRP Kit according to the manufacture’s instruction (Southern Biotech Co., LTD, Birmingham, USA). Microtiter plates were coated with capture antibody (10 μg/ml; provided by the commercial Kit) in 100 μl of phosphate buffered saline (PBS; pH7.4) at 4°C overnight. Plates were washed twice with PBS plus 0.05% Tween20 (PBS-T) and blocked with PBS containing 1% BSA (PBS-1% BSA) for 1 h at room temperature. After being washed with PBS-T, the wells were incubated with 100 μl of horseradish-peroxidase (HRP) conjugated anti-mouse IgG (diluted in 1:250 in PBS-1% BSA), anti-mouse IgG1 or IgG2a (1:500) at 37°C for 60 min. After incubation with 100 μl substrate solution (pH4.0) (1.05% citrate substrate buffer; 1.5% ABTS; 0.03% H_2_O_2_) for 20 min, the absorbance was measured at 405 nm using an ELISA reader (Bio-TekEL × 800, USA). All samples were run in triplicate.

### Lymphocyte proliferation assays

Two weeks after the final immunization, splenocytes were harvested from 3 mice of each group as described previously [21]. After the erythrocytes were lysed using erythrocyte lysis buffer (0.15 M NH_4_Cl, 1.0 M KHCO_3_, 0.1 mM EDTA, pH 7.2), the splenocytes were resuspended in DMEM medium supplemented with penicillin, streptomycin and 10% fetal calf serum (FCS). The cells were counted with a haemocytometer by trypan blue dye exclusion technique and distributed at a density of 5 × 10^5^ cells in 96-well costar plates with stimulated by TLA (10 μg/ml), concanavalin A (ConA; 5 μg/ml; Sigma; positive control) or medium alone (negative control) at 37°C in a 5% CO_2_. The proliferative activity was measured using a 3-(4,5-dimethylthylthiazol-2-yl)-2,5-diphenyltetrazolium bromide (MTT, 5 mg/ml, Sigma) dye assay, as previously described [22]. The stimulation index (SI) was calculated as the ratio of the average OD_570_ value of wells containing TLA-stimulated cells (OD_570TLA_) to the average OD_570_ value of wells containing only cells with medium (OD_570M_). All experimental and control samples were run in triplicate.

### Cytokine assays

Splenocytes from each group were harvested and cultured with TLA in flat-bottom 96-well microtiter plates as described for the lymphocyte proliferation assay. Culture supernatants were harvested and detected for IFN-γ at 96 h, IL-2 at 24 h, IL-4 at 24 h and IL-10 at 72 h according to a protocol of the manufacture’s instruction. Cytokine concentrations were determined using commercial ELISA kits (Biolegend, USA) by reference to standard curves constructed with known amounts of mouse recombinant IFN-γ, IL-2, IL-4 and IL-10. The sensitivity limits for the assays were 8.0 pg/ml for IFN-γ, 0.9 pg/ml for IL-2, 0.5 pg/ml for IL-4, 23.8 pg/ml for IL-10, respectively. The analysis was performed with the data from three independent experiments.

### Cell surface staining of splenic lymphocytes

After viability determination of harvested lymphocytes from each group using 0.04% trypan blue (viability > 90%) and adjusted cell concentration to 1 × 10^6^ cells/ml in PBS containing 2% FBS, the cells were incubated with surface markers including phycoerythrin (PE)-labeled anti-mouse CD3, Allophycocyanin (APC)-labeled anti-mouse CD4 and fluorescein isothiocyanate (FITC)-labeled anti-mouse CD8 (eBioscience) at 4°C for 30 min in the dark. Followed by washed by 2 ml PBS and then fixed with FACScan buffer (PBS containing 1% FCS and 0.1% Sodium azide) and 2% paraformaldehyde, the cultures were analyzed of fluorescence profiles on a FACScan flow cytometer (BD Bio-sciences) by SYSTEM II software (Coulter).

### Statistical analysis

All statistical analyses were performed by SPSS13.0 Data Editor (SPSS Inc., Chicago, IL, USA). The differences of the data (e.g. antibody responses, lymphoproliferation assays and cytokine production) between all the groups were compared by one-way ANOVA. The results in comparisons between groups were considered different if *P* < 0.05.

## Results

### Identification of the expressed product by IFA

Expression of recombinant plasmid (pVAX-CDPK1) in vitro was analyzed by IFA at 48 h post-transfection, and specific green fluorescence was observed in Marc-145 cells transfected with pVAX-CDPK1, whereas no fluorescence was observed in the cells transfected with pVAX I (Additional file 1: Figure S1). The results showed that recombinant TgCDPK1 protein was successfully expressed in Marc-145 cells.

### Humoral response induced by DNA immunization

**Figure 1:**
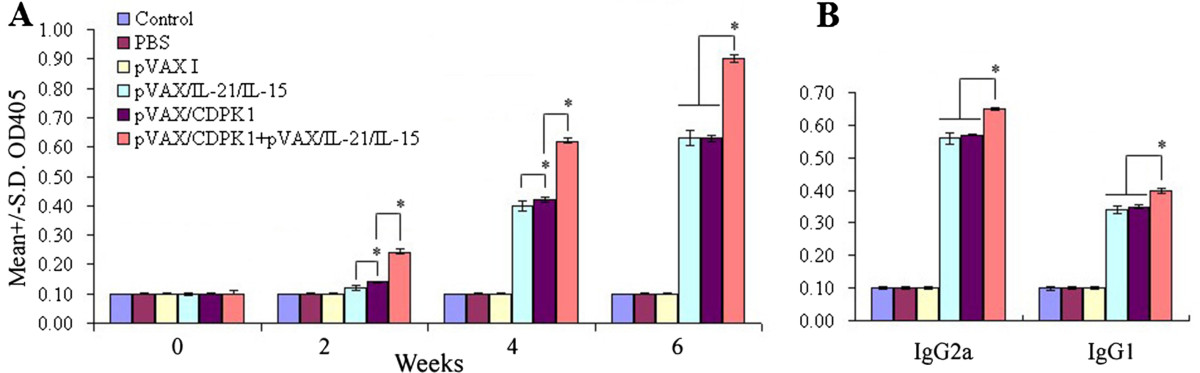
**Humoral response in Kunming mice induced by DNA vaccination. (A)**. Determination of IgG antibodies in the sera of Kunming mice immunized with pVAX-CDPK1, pVAX-IL-21-IL-15, pVAX-CDPK1 + pVAX-IL-21-IL-15, pVAX I, PBS and blank controls on weeks 0, 2, 4, 6. **(B)**. Determination of IgG subclass profiles (IgG1 and IgG2a) in sera of the immunized mice two weeks after the last immunization. Results are expressed as mean of the OD_405_ ± S.E. (n = 3) and statistically significant differences (*P* < 0.05) are indicated by (*).

In order to evaluate the level of antibody induced by DNA immunization, we detected the total IgG followed by three times immunization and thus distribution of IgG1 and IgG2a isotypes two weeks after last immunization. As results, in contrast to PBS, pVAX I, or blank controls, a significant antibody responses corresponding to total antibodies including IgG, IgG1and IgG2a (*P* < 0.05) were induced in immunized mice including the groups of pVAX-CDPK1, pVAX-IL-21-IL-15 and pVAX-CDPK1 plus pVAX-IL-21-IL-15 (Figure 1A and B). The highest antibody levels were observed in co-injection of pVAX-IL-21-IL-15 and pVAX-CDPK1 in mice, and thus the increase of antibody levels occurred with successive DNA immunizations (Figure 1A).

The distribution of IgG1 and IgG2a isotypes was analyzed two weeks after the last immunization. Both IgG1 and IgG2a were detected in the sera of mice immunized with pVAX-CDPK1, pVAX-IL-21-IL-15 and pVAX-CDPK1 plus pVAX-IL-21-IL-15, but a predominance of IgG2a over IgG1 (Figure 1B), but also co-injection of pVAX-IL-21-IL-15 with pVAX-CDPK1 significantly increased the ratio (*P* < 0.05). These results suggest that the specific humoral response and Th1 type immune response were elicited by DNA immunization and further enhanced by co-adminitrsation of plasmids coding for IL-15 and IL-21.

### Cellular immune responses

In order to analyze whether the spleen cells has proliferated, and even produced immunological memory, and thus the percentages of T cell subpopulation after T cell proliferation followed by DNA immunization, we have detected the proliferation Stimulation index (SI) and the percentages of CD4+ and CD8+ T cells in the spleen of immunized mice. As results, SI, in spleen cells from mice immunized with pVAX-CDPK1 or pVAX-IL-21-IL-15 alone was significantly higher in contrast to that with pVAX I and PBS groups, but there was no any significant different among three control groups (*P* > 0.05). While co-inject pVAX-IL-21-IL-15 with pVAX-CDPK1 to mice, the level of splenocyte proliferation was further increased (*P* < 0.05) (Table 1).

**Table 1:** **Cytokine production by splenocytes of immunized Kunming mice after stimulation by** ***Toxoplasma*** **lysate antigen (TLA)**

Group (n = 3)	Cytokine production (pg/ml)				Proliferation (SI)
	IFN-γ	IL-2	IL-4	IL-10	
pVAX-CDPK1 + pVAX-IL-21-IL-15	867.78 ± 15.63^A*^	532.54 ± 23.06^A^	154.44 ± 11.16^A^	131.04 ± 10.31^A^	5.57 ± 0.01^A^
pVAX-IL-21-IL-15	505.12 ± 32.72^B^	314.77 ± 27.06^B^	105.15 ± 16.82^B^	96.54 ± 20.03^B^	3.79 ± 0.05^B^
pVAX-CDPK1	563.65 ± 12.97^C^	353.12 ± 10.93^C^	111.94 ± 8.90^B^	109.72 ± 8.65^B^	4.19 ± 0.14^C^
pVAX I	53.02 ± 3.73^D^	52.04 ± 1.43^D^	51.01 ± 1.08^C^	51.13 ± 1.70^C^	1.05 ± 0.00^D^
PBS	52.92 ± 2.73^D^	51.49 ± 2.67^D^	49.84 ± 1.37^C^	50.20 ± 1.24^C^	1.04 ± 0.07^D^
Control	52.53 ± 2.43^D^	51.56 ± 1.43^D^	49.50 ± 1.79^C^	49.75 ± 1.16^C^	1.03 ± 0.03^D^

*SI* stimulation index.

Splenocytes from 3 mice were harvested 2 weeks after the last immunization.

Values for IFN-γ are after 96 h, values for IL-2 and IL-4 are after 24 h, and values for IL-10 are after 72 h.

*The same superscript letter “D” on the shoulders of experimental data means no statistically significant difference (*P* > 0.05) among different experimental groups from the same measurement, while different letter including A, B, C means statistically significant difference among different experimental groups from the same measurement (*P* < 0.05).

**Figure 2:**
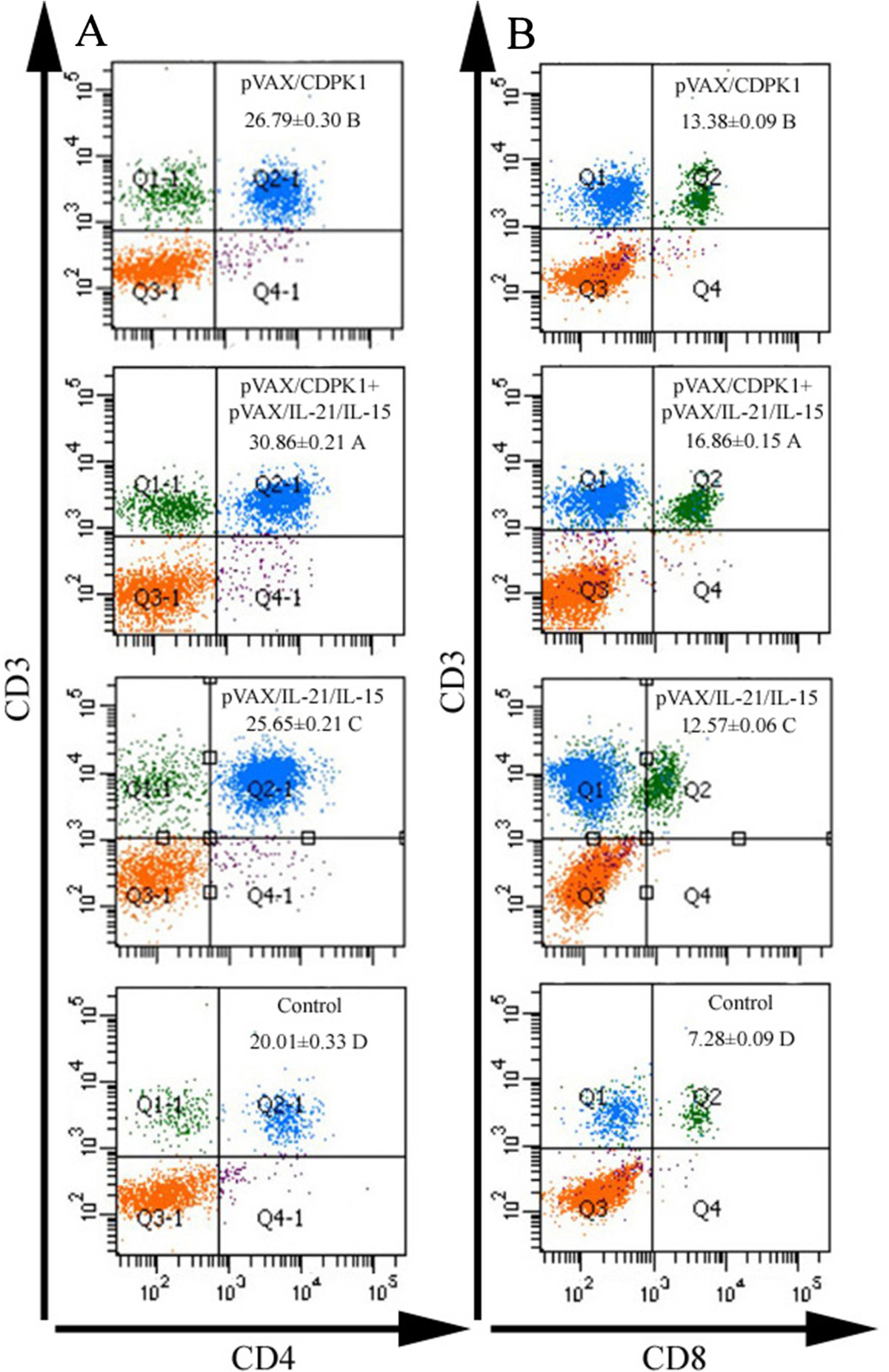
**Detection of lymphocyte subpopulations using fluorescence assisted flow cytometry (FACS). (A)** The percentages of CD3+ CD4+ CD8-T lymphocytes (CD3 gated) in mice spleen cells. **(B)** The percentages of CD3+ CD8+ CD4-T lymphocytes (CD3 gated) in mice spleen cells.

T cell mediated cellular immune response after DNA vaccination were analyzed using flow cytometry analysis of the percentages of CD4+ and CD8+ T cells in the spleen of immunized mice. As depicted in Figure 2, the percentage of CD3 + CD8 + CD4-T cells and CD3 + CD4 + CD8-T cells were significantly increased in pVAX-CDPK1 or pVAX-IL-21-IL-15 immunized mice compared with PBS, pVAX I or the blank control groups, and thus the most increase presented in pVAX-CDPK1 plus pVAX-IL-21-IL-15. There was no significant different between three control groups (*P* > 0.05).

### Production of cytokines by spleen cells

In order to analyze the type and level of T cell response, we detected the levels of Th1 and Th2 type cytokines. As results, a significant increase in secreted IFN-γ and IL-2 were detected in mice immunized with pVAX-CDPK1 or pVAX-IL-21-IL-15 alone (Table 1), and the largest amounts of both two cytokines were produced in mice immunized with pVAX-CDPK1 plus pVAX-IL-21-IL-15 in contrasted with that in the pVAX I, PBS and the blank controls (*P* < 0.05). Furthermore, small amounts of IL-4 and IL-10 were also secreted from splenocytes stimulated by TLA in vitro in all immunized mice compared to the control groups (*P* < 0.05).

### Assessment of protective efficacy of DNA immunized mice against *T. gondii*

**Figure 3:**
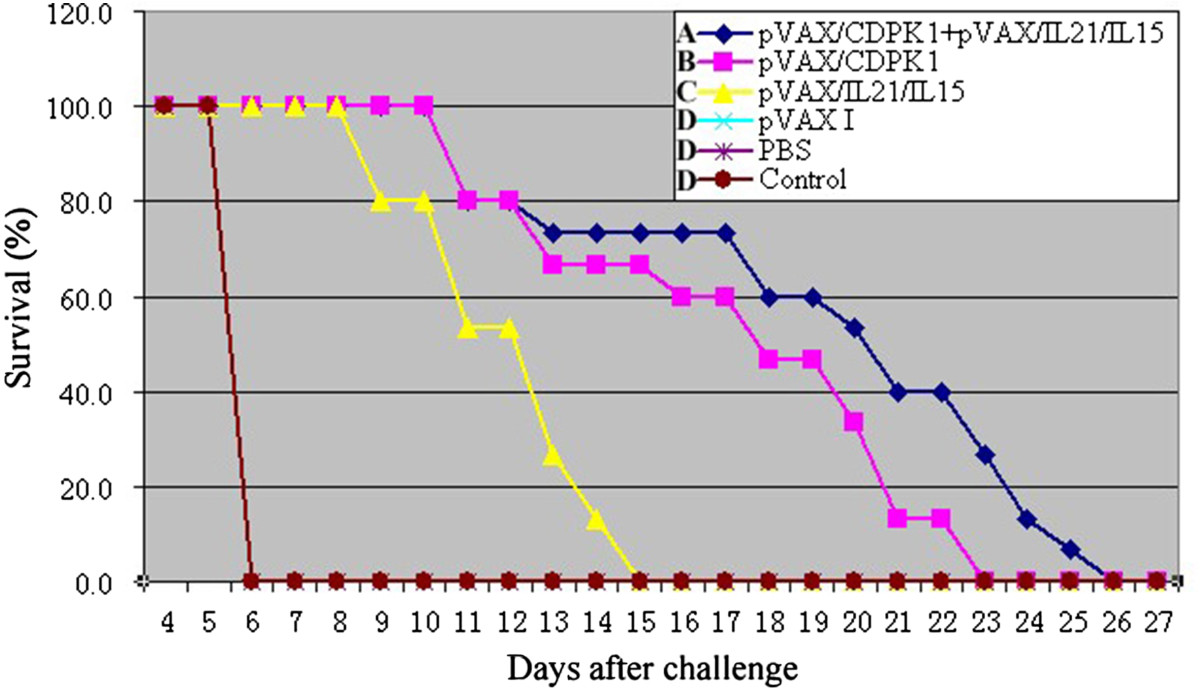
**Protection of Kunming mice against** ***T. gondii*** **infection.** Survival curves of mice immunized with pVAX-CDPK1 (17.3 ± 4.3 days), pVAX-IL-21-IL-15 (12.0 ± 2.0 days), pVAX-CDPK1 + pVAX-IL-21-IL-15 (19.2 ± 5.1 days), pVAX I, PBS and blank controls after lethal challenge with 1 × 10^3^ tachyzoites of virulent *T. gondii* RH strain 2 weeks after the last immunization. Each group had 15 mice. Three control groups (PBS, pVAX I and blank control) had 0% survival at day 6. The same letter in front of experimental group means no statistically significant difference (*P* > 0.05) between different experimental groups from the same measurement, while different letter means statistically significant difference (*P* < 0.05).

In order to analyze the protective efficacy of DNA immunized mice against *T. gondii*, we assess the survival time after acute infection with 10^3^ tachyzoites of the virulent RH strain, and brain cysts after chronic infection with 10 PRU strain. As results, survival curves of different groups of mice are shown in Figure 3. Mice immunized with pVAX-CDPK1 (17.3 ± 4.3 days) or pVAX/IL-21/IL-15 (12.0 ± 2.0 days) significantly prolonged survival time after challenge with 10^3^ tachyzoites of the virulent RH strain in comparison to mice in groups of pVAX I, PBS and blank control, but there was significant difference of survival time between the group of pVAX-CDPK1 and pVAX/IL-21/IL-15 (*P* < 0.05). Co-administration with pVAX-CDPK1 and pVAX/IL-21/IL-15 enhanced the survival time of the immunized mice (19.2 ± 5.1 days) in contrast to the group of pVAX-CDPK1 or pVAX/IL-21/IL-15 (*P* < 0.05). All control mice died within 6 days after challenge, and no significant difference was observed among these control groups (*P* > 0.05). Similar to the results of challenge with RH strain, the immunized groups challenged with PRU strain cysts showed a significant reduction in the number of cysts in the brain (*P* < 0.05) (Table 2).

**Table 2:** **Mean cyst burden per mouse brain 4 weeks after challenge with 20 cysts of** ***Toxoplamsa gondii*** **strain PRU per mouse**

Group (n = 3)	No. of brain cysts (Means ± S.D.)	% reduction*
Control	3117 ± 140^A**^	-
PBS	3067 ± 125^A^	1.6
PVAX I	3067 ± 125^A^	1.6
pVAX-CDPK1	1758 ± 150^B^	45.7
pVAX-IL-21-IL-15	1692 ± 111^B^	43.6
pVAX-CDPK1 + pVAX-IL-21-IL-15	850 ± 104^C^	72.7

*The reduction of brain cysts were from the values for the blank control group.

**The same superscript letter means no statistically significant difference (*P* > 0.05) between different experimental groups, whereas different superscript letters mean statistically significant difference among different experimental groups from the same measurement (*P* < 0.05).

^A^ The superscript letter A means no statistically significant difference (*P* > 0.05) between the groups of pVAX I, PBS and control.

^B^ The superscript letter B means no statistically significant difference (*P* > 0.05) between the groups of pVAX-CDPK1 and pVAX-IL-21-IL-15, but it means significant differences of the No. of brain cysts in these two groups in contrast to the control group.

^C^ The superscript letter C means significant differences of the No. of brain cysts in the group of pVAX-IL-21-IL-15 + pVAX-CDPK1 in contrast to the groups of pVAX-CDPK1 and pVAX-IL-21-IL-15 (*P* < 0.05).

## Discussion

Despite some plasmids DNA expressing MIC13, GRA7, IMP1, and eIF4A have been demonstrated to be vaccine candidates against *T. gondii* infection [23–26], the constructs have not been able to induce complete protection and new vaccine constructs using both multiple antigens but also new adjuvants need to be identified. In the present study, an eukaryotic plasmid expressing TgCDPK1 was evaluated against *T. gondii* infection in Kunming mice model. The results showed that intramuscular immunization with pVAX-CDPK1 could elicit specific humoral and cellular immune responses, resulting in partial protective immunity against *T. gondii* RH (type I) and PRU (type II) strains challenge, associated with an increased survival time (17.3 ± 4.3 days, *P* < 0.05) and 45.7% cyst reduction significantly (*P* < 0.05) in contrast with the control mice. Our unpublished data showed that the CDPK1 gene is conserved between Type I and Type II *T. gondii* strains, which is expressed in the bradyzoite as well as the tachyzoite stage. Therefore, we expect that the immune respose against CDPK1 will provide cross-protection between different *T.gondii* genotypes.

Protective immunity against *T. gondii* is highly dependent on CD8+ T cells mediated immune responses, but also effective cellular Th1-biased response and antibodies served as mediators of protection [27–30]. In this study, we first measured humoral immune responses association with the IgG and IgG subclass induced by DNA immunization with pVAX-CDPK1. Similar to those previous studies [23, 24, 31], vaccination with pVAX-CDPK1 induced high specific antibody titers in sera by ELISA assay, and thus elicited the ratio of IgG2a to IgG1 titers, indication of a mixed Th1/Th2 response.

Both IFN-γ and IL-2 secretion were significantly increased in splenocytes from pVAX-CDPK1 immunized mice and low levels of IL-4 and IL-10 was also produced in contrast with the controls, demonstrating a Th1-biased cellular immune response, but also emphasized again the activation of an appropriate T helper response with high levels of Th1 and low levels of Th2 type cytokines could be contributed to prevent CD4+ T cell-mediated severe immunopathology during the acute and chronic stage of *T. gondii* invasion [30].

It is well established that infection with *T. gondii* naturally drives a potent cellular immune responses, especially T cell immune response that is critical to control the infection in mice model [30, 32]. In this study, we found that cellular immune responses were activated by intramuscularly immunization with pVAX-CDPK1, using the MTT assay. Similar to the results from MIC13 and CDPK3 vaccination [24, 31], DNA immunization with pVAX-CDPK1 induced the significantly increased percentage of both CD4+ and CD8+ T cells in contrast to control groups, suggesting that the DNA vaccination induced the activation of CD4+ and CD8+ T cells, which may contribute to the synergistic effect on the T cell mediated cytotoxic T cell response. Therefore, these results mentioned above have indicated that pVAX-CDPK1 was a good DNA vaccine, eliciting the high levels of humoral and even Th1-biased immune responses, just like our previous studies focused on DNA vaccines including eIF4A [23], MIC13 [24], and CDPK3 [31], but these DNA vaccines using single antigen have not induced complete protection, which could be ascribed to a theory that DNA vaccines based on a single antigen have few lymphocyte binding sites and are restricted largely by the major histocompatibility complex (MHC), leading to mount an effective immune response against *T. gondii* infection difficultly [33].

Nevertheless, it has been recognized that DNA vaccine based on a single antigen can only induce limited immunity to *T. gondii*, but the consequences of effective immune responses to DNA vaccines can be further augmented by co-delivery of cytokine adjuvants [34, 35]. Similar to adjuvants including IL-12, IL-18 and IL-15 [18], the addition of cytokine genes combined with IL-21 and IL-15 can facilitate the efficacy of potential DNA vaccines against infectious disease [19, 20]. Moreover, in our previous studies [21], we have found that the synergy of rIL-15 and rIL-21 genes could augment the efficacy of DNA vaccine in the induction of Th1-bised response, and thus pVAX/mIL-21/mIL-15 alone could induced strong immune responses resulting in protective efficacy. So, we choose pVAX/mIL-21/mIL-15 as an adjuvant in pVAX-CDPK1 DNA vaccine in the present study. In this study, codelivery both IL-21 and IL-15 as an adjuvant in DNA vaccines could significantly enhance the humoral and Th1-driven antigen-specific T helper immune responses induced by pVAX-CDPK1, facilitating protective immunity against toxoplasmosis in the mice model, which is was consistent with the results of previous studies that used both IL-21 and IL-15 as an adjuvant in DNA vaccines [19, 20].

We also found that the administration of pVAX-IL-21-IL-15 alone elicited a considerable non-specific protective immunity equivalent with the levels induced by pVAX-CDPK1, which emphasized again that cytokine adjuvant, pVAX-IL-21-IL-15 could act as immunotherapeutic modulation used for *T. gondii* vaccines [21]. Taken together, the cytokines with the synergy of IL-21 and IL-15 appears to be a broadly effective genetic adjuvant that could be used in DNA vaccines against *T. gondii*, but this combination appears to induce enhanced immune sensitization as adverse effects associated with IL-15 and IL-21 adjuvanted vaccinations or immunotherapies, leading to increased risks of autoimmunity or a variety of immune-mediated problems [36]. Therefore, we need to explore these possibilities in further investigation in detail.

## Conclusion

In summary, our study demonstrates that pVAX-CDPK1 is a novel vaccine candidate, with the ability to elicit humoral and cellular immunity to *T. gondii* infection and induce partial protection after challenge. The use of a plasmid expressing IL-21 and IL-15 as an adjuvant have successfully enhanced the immunoprotective effect of TgCDPK1, and suggest that further studies are warranted to evaluate immune-enhancing effect of these genetic adjuvants (cytokines) in other apicomplexan parasites.

## Electronic supplementary material

Additional file 1: Figure S1: Indirect immunofluorescence (IFA) detection of TgCDPK1 expression in Marc-145 cells 48 h post-transfection. (A) Marc-145 cells were transfected with pVAX-CDPK1; (B) empty vector pVAX I. (TIFF 1 MB)

Below are the links to the authors’ original submitted files for images.

Authors’ original file for figure 1

Authors’ original file for figure 2

Authors’ original file for figure 3
