# Supplementary figures and images for: Protective efficacy of Toxoplasma gondiicalcium-dependent protein kinase 1 (TgCDPK1) adjuvated with recombinant IL-15 and IL-21 against experimental toxoplasmosis in mice
Source: BMC Infect Dis. 2014 Sep 6;14:487. doi: 10.1186/1471-2334-14-487 (PMC4165937; doi:10.1186/1471-2334-14-487)

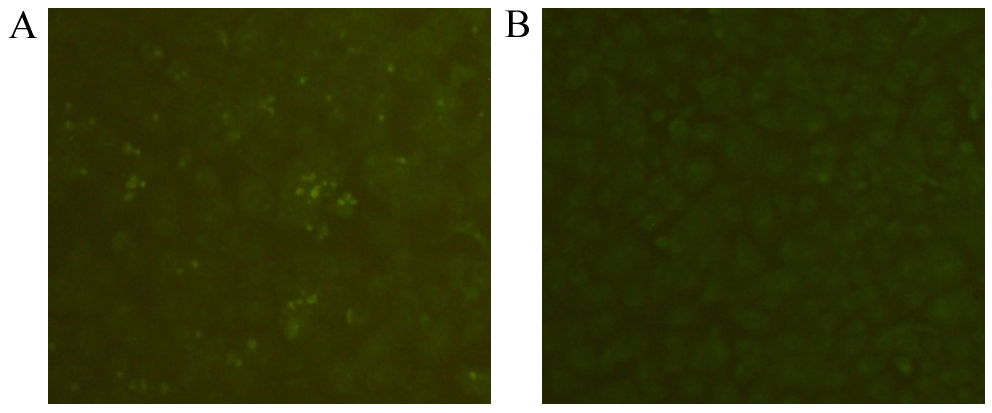

Supplement: Supplementary file 1 — Additional file 1: Figure S1: Indirect immunofluorescence (IFA) detection of TgCDPK1 expression in Marc-145 cells 48 h post-transfection. (A) Marc-145 cells were transfected with pVAX-CDPK1; (B) empty vector pVAX I. (TIFF 1 MB) [file 12879_2014_3793_MOESM1_ESM.tiff]

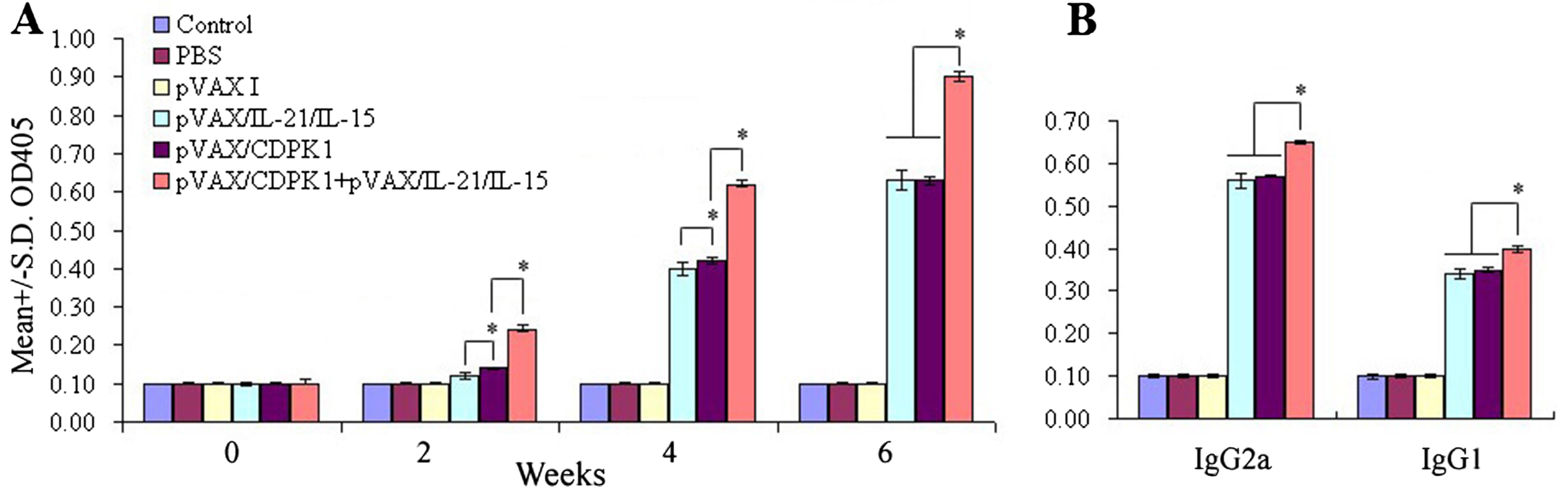

Supplement: Supplementary file 2 — Authors’ original file for figure 1 [file 12879_2014_3793_MOESM2_ESM.tif]

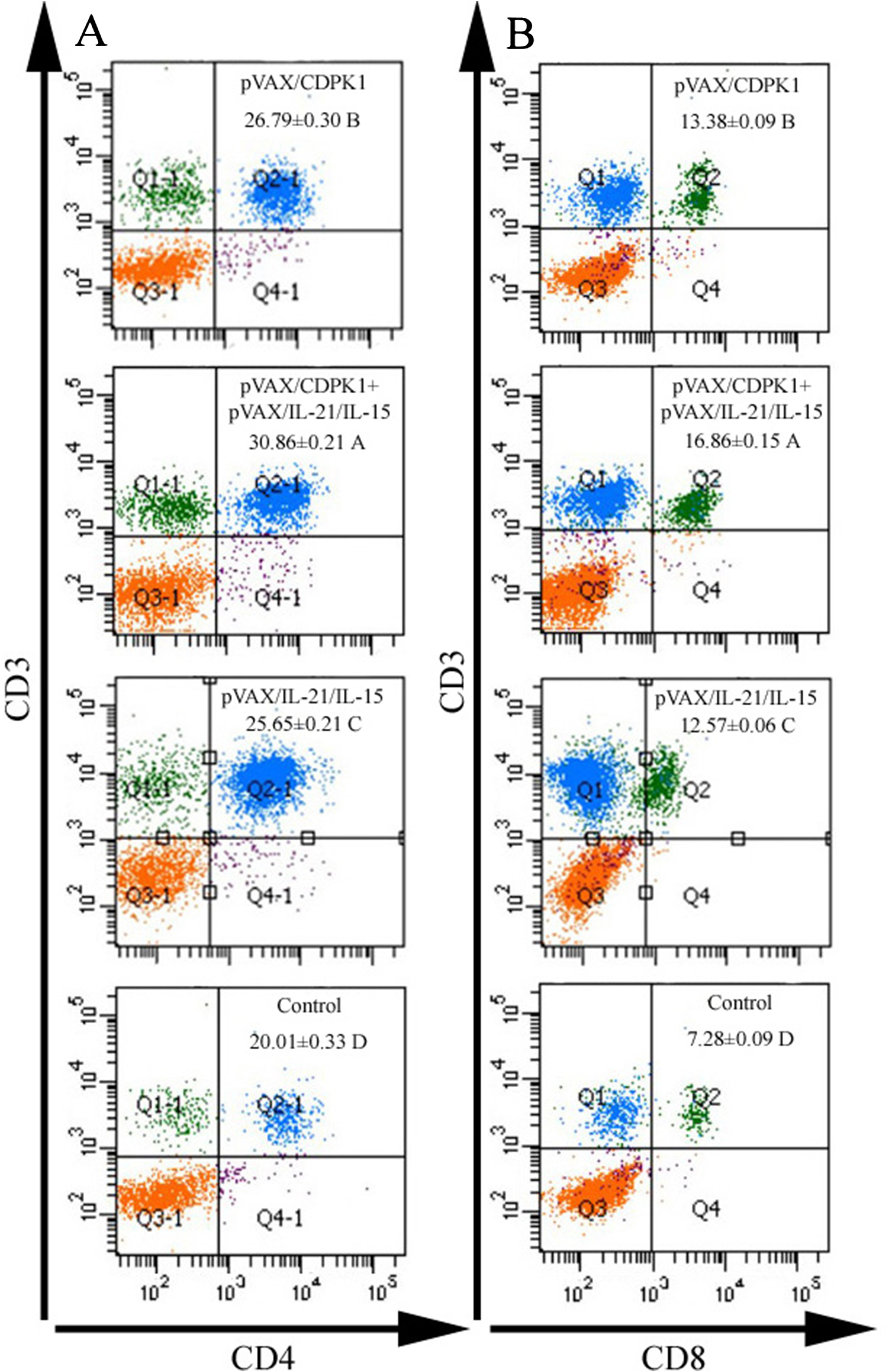

Supplement: Supplementary file 3 — Authors’ original file for figure 2 [file 12879_2014_3793_MOESM3_ESM.tif]

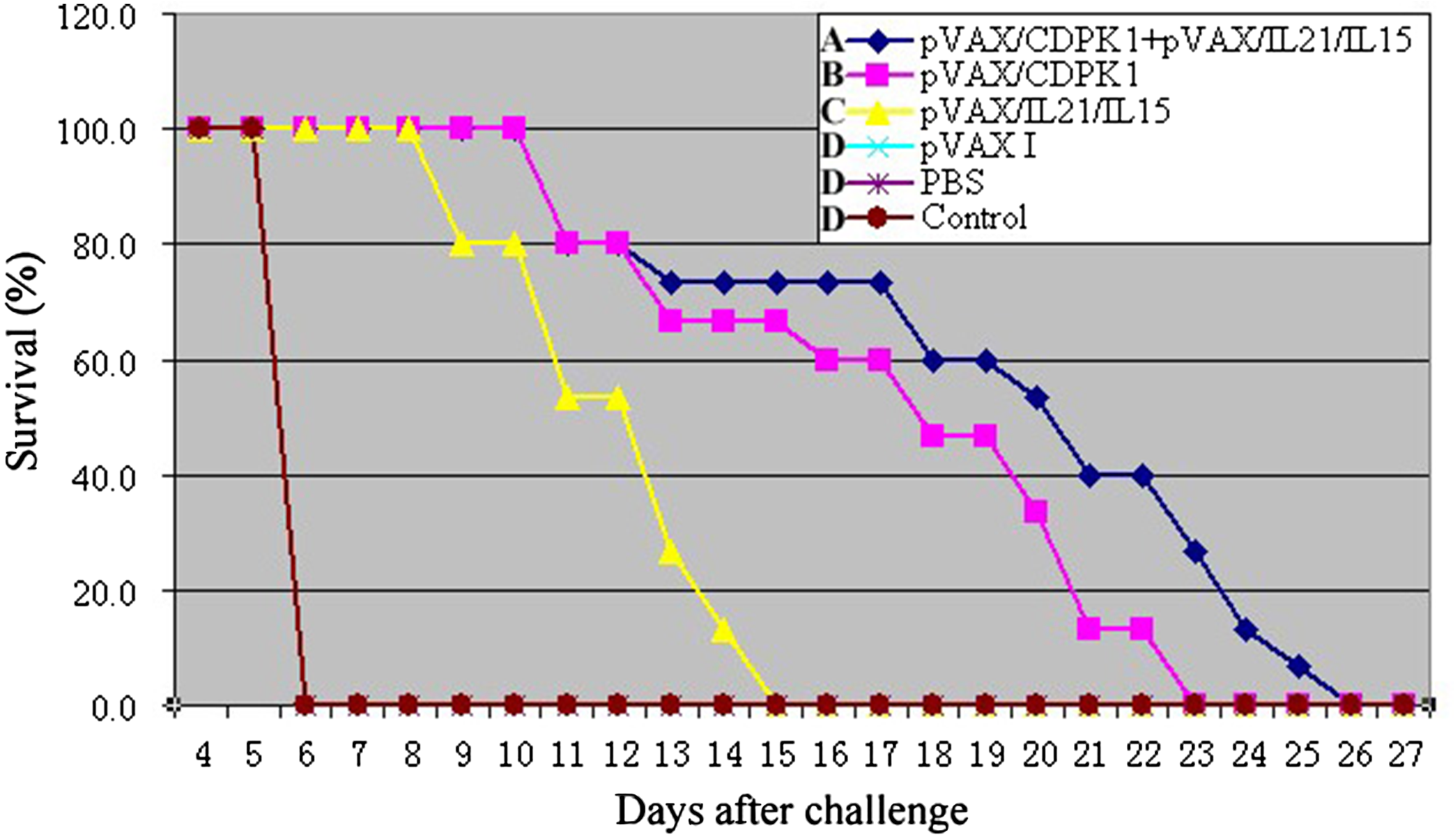

Supplement: Supplementary file 4 — Authors’ original file for figure 3 [file 12879_2014_3793_MOESM4_ESM.tif]
